# Desmoid-Type Fibromatosis of the Male Breast Associated with Bilateral Gynecomastia: Diagnostic Challenges and Surgical Management

**DOI:** 10.3390/jcm15145377

**Published:** 2026-07-09

**Authors:** Tevfik Satir, Uzay Cambaz, Ibrahim Güler

**Affiliations:** 1 Center for Dermatologic Surgery, St. Josefskrankenhaus Heidelberg, Academic Teaching Hospital of the Medical Faculty Mannheim, Heidelberg University, Landhausstrasse 25, 69115 Heidelberg, Germany; 2 Faculty of Medicine, University of Tübingen, Geissweg 5, 72076 Tübingen, Germany; uzay.cambaz@student.uni-tuebingen.de; 3 Department of Plastic, Aesthetic and Hand Surgery, University Hospital Magdeburg, Leipziger Strasse 44, 39120 Magdeburg, Germany; ibrahim.gueler@med.ovgu.de; 4 Department of Health Management, Friedrich-Alexander-Universität Erlangen-Nürnberg FAU, Lange Gasse 20, 90403 Nürnberg, Germany

**Keywords:** breast fibromatosis, desmoid-type fibromatosis, male breast tumor, gynecomastia, breast carcinoma mimic, oncoplastic surgery, case report

## Abstract

**Background/Objectives:** Breast fibromatosis (desmoid-type fibromatosis) is a rare, locally aggressive neoplasm that may closely mimic breast carcinoma on imaging. Occurrence in male patients is exceptionally uncommon, particularly in association with gynecomastia. We report a rare case of male breast fibromatosis and discuss the diagnostic challenges, surgical decision-making, and reconstructive management. **Methods:** A 35-year-old male presented with a palpable right breast mass and bilateral grade II gynecomastia. Histopathological and immunohistochemical analyses were performed. Following multidisciplinary evaluation, the patient underwent wide local excision of the lesion with immediate glandular-adipose tissue rearrangement and simultaneous contralateral gynecomastia correction. **Results:** Ultrasonography demonstrated a hypoechoic lesion of 12 × 11 mm, whereas mammography revealed an irregular mass measuring up to 34 mm. Core needle biopsy showed a fibroblast-rich spindle-cell proliferation with nuclear β-catenin positivity, consistent with desmoid-type fibromatosis. Final histopathology confirmed a 2.5 cm lesion with negative resection margins (minimum margin 5 mm). Immediate reconstruction achieved a satisfactory chest contour and symmetry. No evidence of recurrence was observed during 18 months of follow-up. **Conclusions:** Breast fibromatosis should be considered in the differential diagnosis of suspicious male breast lesions because radiological findings may closely resemble breast carcinoma. Histopathological confirmation remains essential for appropriate management. In selected patients, immediate glandular-adipose tissue rearrangement may facilitate single-stage defect reconstruction and preservation of chest contour following oncologic excision.

## 1. Introduction

Breast fibromatosis (desmoid-type fibromatosis) is a rare fibroblastic neoplasm characterized by infiltrative local growth without metastatic potential. Although desmoid tumors account for approximately 3–4% of soft tissue neoplasms, breast involvement is exceptionally uncommon, representing less than 0.2% of all breast tumors. The disease predominantly affects women in their fourth and fifth decades of life, while occurrence in male patients is exceptional. It is often associated with prior breast trauma or local surgical intervention, and, despite its benign histological appearance, breast fibromatosis may demonstrate locally aggressive behavior, infiltrate surrounding soft tissues, and recur after treatment, which frequently complicates both diagnosis and management [1,2,3,4,5].

The term “desmoid” is derived from the Greek word desmos, meaning “tendon-like,” reflecting the firm consistency of these tumors. It was first introduced by Johannes Müller to describe their characteristic fibrous structure, a finding later confirmed by Nichols in abdominal tumors. Extra-abdominal desmoid tumors are also referred to as deep fibromatosis [6]. Breast fibromatosis originates from fibroblasts and myofibroblasts and is characterized by infiltrative growth into surrounding soft tissues, including the fascia and pectoral musculature. Although histologically benign, the lesion may exhibit locally destructive behavior and significant recurrence rates after surgical excision. Unlike breast carcinoma, distant metastasis does not occur; however, the infiltrative nature of the disease often complicates both diagnosis and surgical management [2,5,6].

While surgical excision has traditionally been a primary treatment modality, recurrence rates remain substantial, highlighting the importance of adequate margins and structured follow-up. Although age and tumor size have been identified as relevant prognostic factors, the impact of margin status on recurrence remains controversial [5,6].

Clinically, breast fibromatosis usually presents as a painless palpable mass and frequently mimics carcinoma on imaging. Mammography may reveal irregular or spiculated high-density lesions, while ultrasonography often demonstrates poorly circumscribed hypoechoic masses. Although magnetic resonance imaging can help evaluate lesion extent and chest wall involvement, definitive diagnosis still relies on histopathological confirmation, as imaging findings are not pathognomonic. The etiology remains incompletely understood; however, previous surgery, trauma, hormonal influences, genetic predisposition, and gynecomastia in male patients have all been proposed as possible contributing factors [1,3,4,5,7].

Given its rarity in male patients and its potential to mimic malignancy, breast fibromatosis presents a diagnostic and therapeutic challenge. Furthermore, the role of reconstructive strategies in this setting has not been sufficiently addressed in the literature. Recent studies have also suggested a shift toward more individualized management approaches, including active surveillance in selected cases [1,2,3,4,5,7].

We report a rare case of desmoid-type fibromatosis of the male breast associated with bilateral gynecomastia that radiologically mimicked breast carcinoma. The case highlights the diagnostic challenges, surgical decision-making process, and reconstructive management following oncologic excision in an exceptionally uncommon clinical scenario.

## 2. Case Presentation

A 35-year-old male presented to our plastic surgery outpatient clinic with a six-month history of a palpable mass in the upper outer quadrant of the right breast, accompanied by bilateral grade II gynecomastia. His medical history was otherwise unremarkable, with no previous breast surgery or trauma and no known familial syndromes, including familial adenomatous polyposis (FAP) or Gardner syndrome. There was no personal or family history of breast cancer or other malignancy. The patient denied nipple discharge, weight loss, or other systemic symptoms.

**Figure 1:**
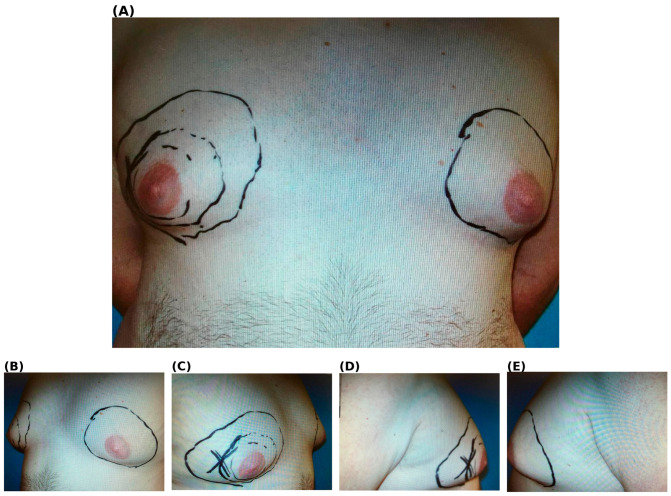
Preoperative clinical appearance with surgical markings, illustrating the bilateral side-specific planning. (**A**) Frontal overview with markings on both sides: planned circumferential excision around the palpable lesion on the right (tumor-bearing) side, and standard gynecomastia correction markings on the contralateral left side. (**B**) Oblique view of the contralateral (left) side. (**C**) Oblique view of the tumor-bearing right side, with the lesion location indicated (X) within the planned excision area. (**D**) Lateral view of the tumor-bearing right side. (**E**) Lateral view of the contralateral left side.

Physical examination revealed a firm, discrete subcutaneous mass measuring approximately 2 cm in diameter in the upper outer quadrant of the right breast. The lesion was painless at rest but tender on direct palpation. No skin retraction, nipple abnormalities, or palpable axillary lymphadenopathy were identified. Bilateral grade II gynecomastia was present (Figure 1).

**Figure 2:**
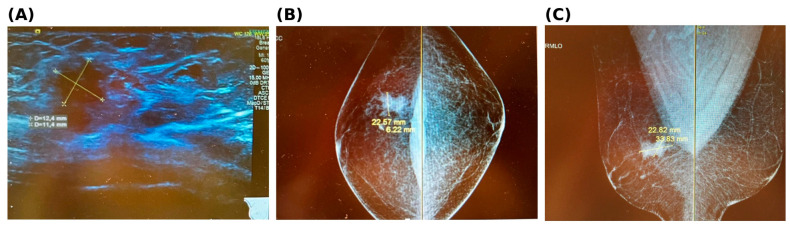
Imaging findings of the right breast lesion. (**A**) Ultrasonography demonstrating an irregular hypoechoic lesion measuring approximately 12.4 × 11.4 mm with poorly defined margins. (**B**) Mammography in the craniocaudal (CC) view, showing a high-density lesion measuring approximately 22.6 mm in greatest in-plane dimension. (**C**) Mammography in the right medio-lateral oblique (RMLO) view, showing the same lesion measuring approximately 33.8 mm in greatest in-plane dimension, corresponding to a maximum mammographic extent of approximately 34 mm.

Ultrasonography demonstrated a poorly defined hypoechoic lesion measuring approximately 12.4 × 11.4 mm within the right breast (Figure 2A). Mammography revealed an irregular high-density mass that appeared highly suspicious for malignancy. The lesion measured approximately 22.6 mm in greatest dimension on the craniocaudal (CC) projection (Figure 2B) and approximately 33.8 mm in greatest dimension on the right medio-lateral oblique (RMLO) projection (Figure 2C), giving a maximum mammographic extent of approximately 34 mm. Because standard mammographic views are two-dimensional projections acquired under breast compression, the in-plane measurements of an ill-defined infiltrative lesion are not truly orthogonal and were not combined into a single three-dimensional size; the maximum in-plane dimension was used to express mammographic extent. The greater extent measured on mammography compared with ultrasonography likely reflects the spiculated, infiltrative growth of the lesion, whose architectural distortion may extend beyond the discrete hypoechoic core delineated on ultrasonography. The contralateral side showed imaging features consistent with gynecomastia. Magnetic resonance imaging was not performed preoperatively because mammography, ultrasonography, and tissue sampling were considered sufficient for treatment planning, and additional MRI findings were not expected to alter management.

Ultrasound-guided tissue sampling was performed using both fine-needle aspiration for cytology and core needle biopsy. Histopathological examination demonstrated a fibroblast-rich spindle-cell proliferation with interspersed collagen fibers and occasional perivascular lymphocytes and plasma cells, with no evidence of epithelial malignancy. Immunohistochemistry revealed nuclear β-catenin expression and positivity for smooth muscle actin, while staining for CD34, CD117, and estrogen receptor was negative. These findings were consistent with desmoid-type fibromatosis.

A multidisciplinary review was conducted prior to surgery. Although active surveillance has become an accepted management option for selected desmoid tumors, surgical treatment was considered appropriate because the lesion was symptomatic and the patient, after discussion of this option, preferred definitive excision, which additionally allowed simultaneous correction of the symptomatic bilateral gynecomastia; although the core biopsy already indicated desmoid-type fibromatosis, excision provided definitive histopathological confirmation.

### 2.1. Surgical Technique

**Figure 3:**
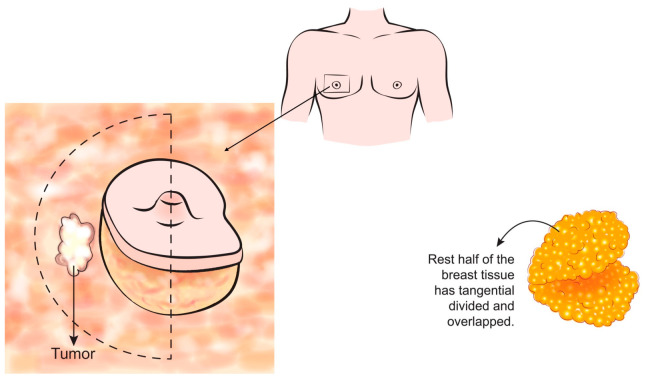
Schematic illustration of the surgical concept on the tumor-bearing side. After periareolar access, the lateral half of the breast tissue containing the lesion is resected en bloc with a surrounding margin of glandular and adipose tissue (**left** panel). The remaining medial half is then tangentially divided and folded as a glandular-adipose flap, advanced and overlapped into the defect (**right** panel), restoring chest contour without prosthetic material or an additional donor site.

On the tumor-bearing right side, a periareolar incision was used. After elevation of the skin flaps, the lateral half of the breast tissue containing the lesion was dissected and removed en bloc with a surrounding margin of glandular and adipose tissue to achieve clear margins. The specimen included the underlying pectoral fascia at the site of closest tumor proximity. The remaining medial half of the breast tissue was then tangentially divided and folded as a glandular-adipose flap, advanced and overlapped into the lateral defect to restore chest contour without prosthetic material or an additional donor site. The nipple–areola complex was preserved and repositioned to maintain symmetry. The surgical concept on the tumor-bearing side is illustrated in Figure 3.

On the contralateral left side, standard gynecomastia correction was performed using liposuction combined with subcutaneous glandular excision through a periareolar approach, allowing volume reduction and contour optimization to match the operated side.

### 2.2. Pathology

**Figure 4:**
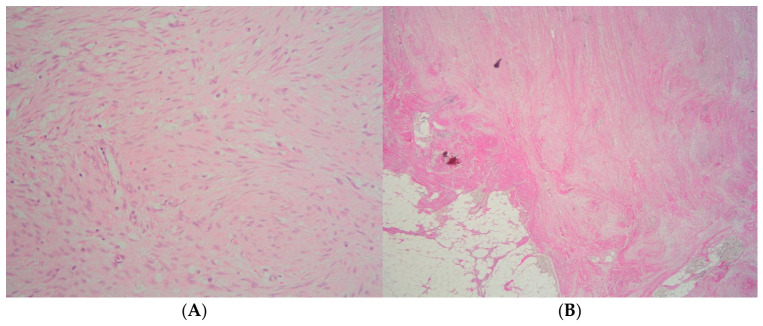
Histopathological findings of desmoid-type fibromatosis. (**A**) Higher-magnification hematoxylin–eosin stain showing bland spindle-cell proliferation embedded within abundant collagenous stroma, without significant cytologic atypia. (**B**) Low-power Elastica–van Gieson (EVG) stain demonstrating a collagen-rich fibroblastic lesion with infiltrative growth into the surrounding adipose tissue.

Final histopathological evaluation confirmed desmoid-type fibromatosis measuring 2.5 cm in greatest dimension. The resected specimen weighed 78 g. Histology demonstrated a fibroblast-rich spindle-cell proliferation with interspersed collagen bundles and low mitotic activity. Immunohistochemical analysis confirmed nuclear β-catenin positivity. Surgical margins were free of tumor, corresponding to an R0 resection, with a minimum margin of 5 mm (Figure 4).

### 2.3. Postoperative Course and Follow-Up

**Figure 5:**
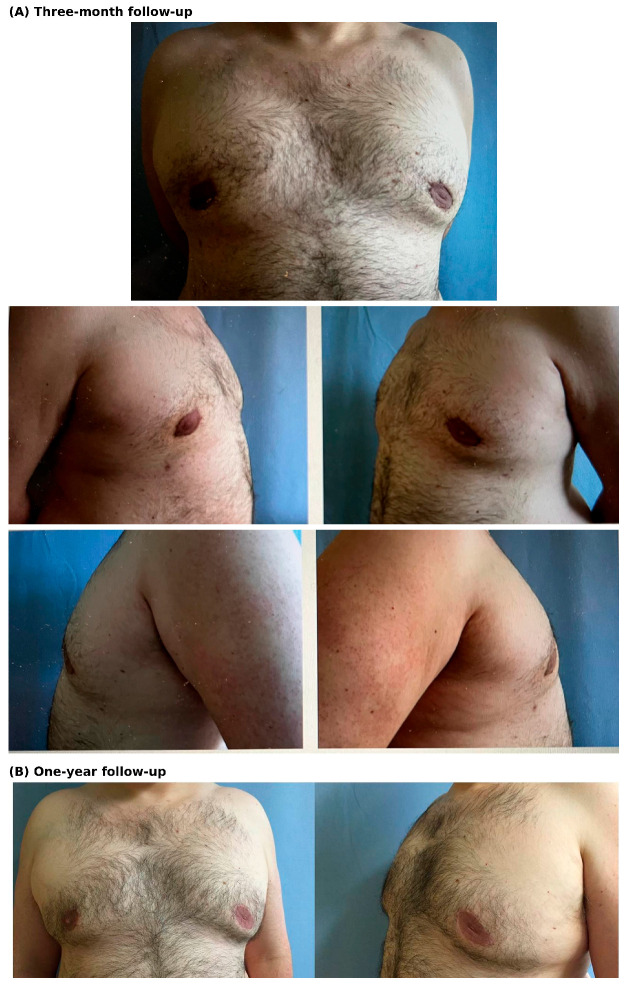
Postoperative clinical appearance demonstrating restored chest contour and bilateral symmetry with inconspicuous periareolar scars. (**A**) Three-month follow-up: frontal view (**top**), oblique views from both sides (**middle**), and lateral views from both sides (**bottom**). (**B**) One-year follow-up: frontal and oblique views, showing a stable, symmetric long-term result.

**Figure 6:**
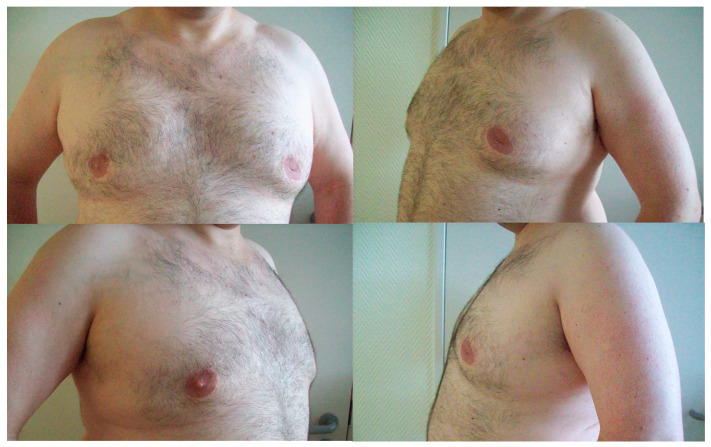
An 18-month postoperative follow-up demonstrating stable chest contour, satisfactory aesthetic outcome, and no clinical evidence of local recurrence. Frontal view (**top left**), left oblique view (**top right**), right oblique view (**bottom left**), and left lateral (90°) view (**bottom right**).

The postoperative course was uneventful. The patient was followed clinically and radiologically. At three-month follow-up, favorable chest contour and bilateral symmetry were observed from frontal, oblique, and lateral views, with well-healed periareolar scars. At one-year follow-up, the result remained stable, with no clinical or radiologic evidence of recurrence (Figure 5). At the most recent review, 18 months postoperatively, the chest contour and symmetry remained stable with no clinical evidence of local recurrence (Figure 6). Continued surveillance has been planned given the recognized potential for late recurrence in desmoid-type fibromatosis.

## 3. Discussion

Breast fibromatosis is a rare entity that frequently mimics carcinoma both clinically and radiologically, often leading to a high index of suspicion for malignancy. Spiculated masses on mammography and irregular hypoechoic lesions on ultrasound are well-described imaging findings that can be difficult to distinguish from invasive breast carcinoma [7,8,9]. In the present case, both ultrasonography and mammography demonstrated suspicious features, further emphasizing the diagnostic challenge posed by this uncommon lesion in a male patient.

Clinically, breast fibromatosis typically presents as a firm, painless mass without the nipple discharge or regional lymphadenopathy more characteristic of carcinoma. On ultrasonography, lesions are commonly described as irregular, hypoechoic masses with parallel orientation, generally lacking the echogenic halo or microcalcifications seen in carcinoma. Mammography typically shows spiculated lesions that may be indistinguishable from malignancy, while magnetic resonance imaging can help delineate tumor extent and chest wall involvement for surgical planning. Definitive diagnosis nevertheless requires histopathological confirmation [7,8,9]. In our patient, the radiological findings were highly suspicious for malignancy despite the benign nature of the lesion.

The discrepancy between imaging and pathological tumor dimensions in the present case is another noteworthy finding. Ultrasonography demonstrated a lesion of approximately 12 mm, mammography an extent of up to 34 mm, and final histopathology a tumor diameter of 25 mm. Rather than indicating that one modality is inherently more accurate, this variability reflects the infiltrative, poorly circumscribed growth of desmoid-type fibromatosis: ultrasonography tends to resolve only the discrete hypoechoic core and therefore underestimates the lesion, whereas the spiculation and architectural distortion seen on mammography extend beyond the tumor and may overestimate it, with the histopathological diameter lying between the two. Magnetic resonance imaging, which was not performed in the present case, is generally regarded as the most reliable modality for delineating true tumor extent [3,4]. Awareness of these inter-modality differences remains important when assessing tumor extent preoperatively.

Desmoid-type fibromatosis may arise in any region of the breast but is frequently located near the pectoralis major muscle and may originate from the underlying pectoral fascia. Clinically, patients may remain asymptomatic for prolonged periods or present with a firm, painless mass. Depending on its origin and extent, the lesion can appear fixed to the chest wall. In some cases, associated skin thickening or retraction may further contribute to its resemblance to malignant breast lesions [7,8].

The etiology of breast fibromatosis remains incompletely understood. Previous studies have suggested possible associations with prior surgery, trauma, silicone implants, hormonal influence, and genetic predisposition (e.g., Gardner syndrome and familial adenomatous polyposis) as contributing factors. In men, gynecomastia reflects a relative excess of estrogen over androgen; whether this hormonal environment also contributes to a coexisting desmoid-type fibromatosis is unclear, and the estrogen-receptor-negative immunoprofile observed in the present case does not support a direct estrogen-driven mechanism in the tumor. No endocrine abnormality was clinically apparent, and no dedicated hormonal evaluation was undertaken. The diagnosis was based on the characteristic histopathological appearance together with nuclear β-catenin expression, a well-recognized feature of desmoid-type fibromatosis; although CTNNB1 mutational analysis was not performed, the histological and immunohistochemical findings were considered sufficient to establish the diagnosis. No personal or familial features suggestive of familial adenomatous polyposis or Gardner syndrome, and no history of prior breast surgery or trauma, were identified. The concurrent bilateral gynecomastia and desmoid-type fibromatosis are therefore best regarded as an incidental association rather than evidence of a shared hormonal cause [1,5,10].

In male patients, breast fibromatosis is often not initially considered, yet its radiologic resemblance to carcinoma may lead to unnecessarily aggressive management. Awareness of this entity, combined with histopathological confirmation, is therefore essential to avoid overtreatment.

The management of desmoid-type fibromatosis has evolved considerably in recent years. Wide local excision with negative margins was historically regarded as the standard treatment strategy due to the locally aggressive behavior and relatively high recurrence rates reported in the literature, ranging from 20–30% [5,10,11]. However, recent international consensus recommendations and contemporary studies suggest a gradual shift toward more individualized management strategies, including active surveillance in selected cases, as recurrence rates after surgery may not differ significantly from those observed with conservative approaches [1,2,12,13]. In addition, non-surgical treatment options, including radiotherapy, hormonal therapy, and targeted systemic treatments, may be considered in selected patients, particularly in cases of unresectable disease, recurrence, or when surgery would result in substantial morbidity. Given the potential for recurrence, structured postoperative follow-up is recommended, especially in the first few years after treatment, when the risk of recurrence appears to be highest [1,4,13].

In the present case, although the core biopsy had already established a benign desmoid-type lesion, definitive excision was preferred over active surveillance because the lesion was symptomatic, the patient wished for definitive treatment, and the coexisting symptomatic bilateral gynecomastia could be addressed in the same procedure. Following multidisciplinary evaluation, wide local excision with immediate reconstruction was considered the most appropriate strategy.

In addition to oncologic control, reconstructive considerations are particularly important in young patients and in cases involving cosmetically sensitive regions [10,11,12,14]. In male patients, even relatively small volume deficiencies may become highly visible because of the relatively flat contour of the male thorax. Therefore, reconstructive planning should be considered an integral component of surgical management rather than a secondary aesthetic step. Although glandular-adipose tissue rearrangement represents a well-established volume-displacement principle in oncoplastic breast surgery, its application in male breast fibromatosis has been only rarely described. In the present case, adaptation of this established reconstructive concept allowed single-stage restoration of chest contour while avoiding secondary deformity and additional donor-site morbidity.

Immediate glandular-adipose tissue rearrangement following tumor excision enabled restoration of chest contour and avoidance of secondary deformity in a single-stage procedure. Simultaneous correction of the contralateral gynecomastia further contributed to postoperative symmetry and overall aesthetic balance. In selected patients, immediate reconstruction may serve as a practical adjunct to oncologic resection, preserving thoracic contour and reducing the need for secondary corrective procedures.

Male breast fibromatosis remains exceptionally rare, and its coexistence with gynecomastia has been documented only exceptionally [3,4,15,16]. The reported cases of desmoid-type fibromatosis of the male breast, together with the present case, are compared in Table 1. Compared with previously reported cases, our patient was relatively young and presented without identifiable predisposing factors such as prior breast surgery, trauma, or known familial syndromes, while imaging findings were highly suspicious for malignancy despite the benign nature of the lesion. This case, therefore, highlights the importance of considering fibromatosis in the differential diagnosis of suspicious male breast lesions, to avoid overtreatment and unnecessary radical procedures. From a surgical perspective, the principal contribution of the present case is that the surplus glandular-adipose tissue created by the coexisting gynecomastia can itself be redeployed as a local volume-displacement flap, allowing oncologic resection, immediate defect reconstruction, and contralateral symmetrization to be achieved in a single stage without implants or a separate donor site. Whereas the reported male cases were managed by partial mastectomy or mastectomy, this combined single-stage oncoplastic approach has rarely been described in the male breast [3,4,15,16].

Based on our management of this case, several practical points may guide surgeons faced with a similar presentation.

First, histological confirmation by core-needle biopsy, including nuclear β-catenin staining, should precede any definitive procedure, because the imaging appearance is indistinguishable from carcinoma and overtreatment must be avoided.

Second, the imaging modalities diverge, with ultrasonography tending to underestimate the infiltrative margins and mammography to overestimate them; no single modality should therefore be relied upon for surgical planning, and magnetic resonance imaging most reliably delineates true extent when required. The pectoral fascia should be included in the deep margin when the lesion abuts it.

Third, because margin status has only a limited influence on recurrence in desmoid-type fibromatosis, wide margins should not be pursued at the expense of a large deformity. In the male chest, the tissue surplus of a coexisting gynecomastia can instead be redeployed to reconstruct the defect in the same setting. Potential complications include hematoma or seroma, contour asymmetry, and malposition or partial necrosis of the nipple–areola complex; these are minimized by meticulous hemostasis, preservation of a well-vascularized glandular-adipose pedicle, and intraoperative assessment in the upright position. Because most recurrences occur within the first three years, we recommend structured follow-up with clinical examination and ultrasonography every three to six months for the first two to three years and annually thereafter for at least five years, reserving magnetic resonance imaging for equivocal findings.

The principal limitations of this report are the relatively short follow-up period and the absence of an objective, validated assessment of the aesthetic outcome. Although clinical and radiological follow-up extended to 18 months without evidence of recurrence, this interval remains short for a tumor that may recur several years after treatment, and the aesthetic result was evaluated subjectively rather than with a standardized or patient-reported outcome instrument [13]. The absence of recurrence at 18 months should therefore be interpreted cautiously, and continued long-term surveillance has been planned. Additional reports and longer follow-up data are required to better define optimal management strategies and reconstructive approaches for male breast fibromatosis.

In summary, male breast fibromatosis associated with bilateral gynecomastia represents an exceptionally rare clinical entity that may closely mimic breast carcinoma both clinically and radiologically. Histopathological confirmation remains essential for accurate diagnosis. This case highlights both the diagnostic challenge posed by this uncommon lesion and the feasibility of adapting established glandular-adipose volume-displacement principles to preserve chest contour following oncologic resection. Although no recurrence was observed at 18 months, longer surveillance remains necessary because recurrence cannot be excluded after a limited follow-up period.

## 4. Conclusions

Breast fibromatosis should be considered in the differential diagnosis of suspicious breast lesions in male patients, as imaging findings may closely mimic malignancy and definitive diagnosis requires histopathological confirmation. In the present case, oncologic excision combined with immediate glandular-adipose tissue rearrangement allowed tumor removal and preservation of chest contour in a single-stage procedure. This case highlights the feasibility of adapting established volume-displacement principles to an uncommon clinical scenario while maintaining satisfactory aesthetic outcomes. Greater awareness of this rare entity may help avoid overtreatment and support individualized management strategies. Long-term follow-up remains essential because recurrence cannot be excluded after a limited surveillance period.

## Figures and Tables

**Table 1:** Comparison of the present case with recently reported cases of desmoid-type fibromatosis of the male breast.

Study	Age (y)	Side	Gynecomastia	Predisposing Factor (s)	Tumor Size	Management	Margins	Follow-Up; Recurrence
Torres Sousa et al., 2011 [15]	52	Right	No	None identified	28 mm (mammography)	Subcutaneous mastectomy	NR	11 mo; no recurrence
Roman et al., 2019 [16]	66	Right	No	APC and PTEN mutation	79 mm (MRI)	Mastectomy; adjuvant tamoxifen	NR	11 mo; no recurrence
Moussaddykine & Sy N’deye, 2023 [4]	40	Left	Yes (chronic, bilateral)	None identified	41 mm (MRI); 19 mm (mammography)	Left mastectomy	NR	NR
Correa Sandoval et al., 2024 [3]	28	Right	No	Prior thoracic liposuction (aesthetic)	2.3 cm	Partial mastectomy	Clear	24 mo (biannual); no recurrence
Present case, 2026	35	Right	Yes (bilateral, grade II)	None identified	2.5 cm	Wide local excision + immediate glandular-adipose flap + contralateral gynecomastia correction	R0 (minimum margin 5 mm)	18 mo; no recurrence

Abbreviations: mo, months; MRI, magnetic resonance imaging; NR, not reported; R0, microscopically negative resection margin; y, years.

## Data Availability

All relevant data are included in this article. Further information is available from the corresponding author upon reasonable request.
